# Synthesis and characterization of salen-Ti(IV) complex and application in the controllable polymerization of D, L-lactide

**DOI:** 10.1371/journal.pone.0201054

**Published:** 2018-08-02

**Authors:** Xiang Li, Baojun Yang, Huaili Zheng, Pei Wu, Guoming Zeng

**Affiliations:** 1 School of Civil Engineering and Architecture, Chongqing University of Science and Technology, Chongqing, China; 2 Key Laboratory of the Three Gorges Reservoir Region’s Eco-Environment, State Ministry of Education, Chongqing University, Chongqing, China; 3 Chongqing Huashu Robotics Co., Ltd., Chongqing, China; Brandeis University, UNITED STATES

## Abstract

Poly(lactic acid) has been extensively investigated in the biomedical field because of its good biocompatibility and biodegradability. As an important method of poly(lactic acid) synthesis, metal complex-catalyzed ring-opening polymerization (ROP) of lactide can achieve a controllable lactide polymerization through the selection of appropriate ligands and metals. In this study, a novel metal (LTi–O)_2_ complex was synthesized and structurally characterized. (LTi–O)_2_ showed a relatively high catalytic activity and controllability of Poly(D, L-lactide) (PDLLA) molecular weights (polydispersity index of 1.02–1.22) in the ROP of D,L-lactide. The kinetic equation of D,L-LA ROP catalyzed by (LTi–O)_2_ could be expressed as–*d*[M]/*dt* = *k*[M]^2^[(LTi–O)_2_]^1^, and the reaction activation energy was 95.67 kJ·mol^−1^. Physical/chemical properties and biocompatibility evaluation results showed that PDLLA obtained through the (LTi–O)_2_-catalyzed ROP of D,L- lactide exhibited a good degradation performance and excellent biocompatibility.

## Introduction

Polylactide (PLA) is a hydrolysable aliphatic polyester that has been used in packaging materials, surgical sutures, drug delivery systems, and tissue engineering scaffolds because of its good biocompatibility and biodegradability [1–3]. PLA can be prepared through lactic acid polycondensation. However, some drawbacks, such as low-molecular-weight and high-molecular-weight dispersity of PLA, have been obtained through the condensation polymerization route [1,2]. High-molecular-weight PLA is mainly synthesized through the ring-opening polymerization (ROP) of D,L-lactide or L-lactide catalyzed by coordination compounds [1–4]. Therefore, developing novel, effective, cheap, and non-toxic metal catalysts for biodegradable polymer production can help reduce the production cost of polymers and enhance their biochemical properties. ROP catalyzed by a metal complex can effectively control the molecular weight and architecture of a polymer and produce a macromolecular polymer with a narrow molecular weight distribution.

The choice of supporting ligands on central metals is essential for the catalytic behavior of complexes. Therefore, metal complexes with special structures have been widely used. Some metal complexes with aryloxy (imine aryloxy, aryloxy ammonia, salen-bisaryloxy, and half-salen aryloxy) exhibit excellent catalytic activity, and they have been utilized to catalyze lactide ROP [5–8]. During lactide ROP and PLA chain growth catalyzed by these types of metal complexes, side reactions, such as intramolecular and intermolecular transesterification of PLA, are blocked because of the steric effect of ligands with large volumes [4, 6, 8]. Therefore, controllable polymerization is achieved, and polymers with narrow molecular weight distributions are obtained.

In the development of new catalytic systems, an important task is to create biocompatible and less toxic catalysts or initiators that can be used for biomedical applications. Some metal complexes, such as magnesium, calcium, zinc, aluminum, and tin (II), show potential for such applications because of their low toxicity, and they have been reported as effective catalysts for the ROP of lactide [9–12].

Titanium complexes have exhibited a unique activity in producing polyethylene (PE) with controlled molecular weights and microstructures [13, 14]. The catalytic activity of these complexes can promote their further exploration as catalysts for the bulk- and solution-phase polymerization of lactide. Titanium complexes featuring bis(aryloxo), bis(trimethylsilyl) amides, and biphenoxy ligands have catalytic activities for lactide ROP [13–15]. Therefore, other titanium complexes with various salen ligands should be developed for lactide ROP to prepare PLAs with increased molecular weights and narrow molecular weight distributions. A highly active catalyst requires not only a strong coordination ability between a metal atom in the catalyst and a carbonyl oxygen atom in lactide but also the ability to increase the electron density of oxygen atoms in the oxygen–metal bond of the catalyst. Such an increase can enhance the ability to attack carbonyl carbon atoms in lactide. The effects of long-term heating on the decomposition of metal complexes during catalytic polymerization should also be investigated. Therefore, nitrogen atoms with additional electrons can be introduced to a metal complex and coordinated with a metal atom to increase the stability of metal complexes. Considering the presence of side reactions, such as intermolecular and intramolecular ester exchange reactions during lactide ROP, we can also coordinate ligands possessing a large steric hindrance with a metal atom [16]. This type of metal complexes can reduce the incidence of side reactions and consequently decrease the polydispersity index (PDI) of a polymer.

This study aimed to prepare a nontoxic metal complex catalyst with a high catalytic activity and thermal stability. A metal complex named (LTi–O)_2_ was synthesized through a simple condensation and coordination reaction. Fourier transform infrared (FTIR) spectroscopy, ^1^H NMR spectroscopy, and elemental analyses were conducted to determine the structure of (LTi–O)_2_. Thermogravimetric (TG)/differential scanning calorimetry (DSC) analysis was performed to evaluate its thermal stability. The ROP catalytic activity of (LTi–O)_2_ was examined, and the kinetics of ROP was reported in detail. Finally, the physical/chemical properties and biocompatibility of PDLLA obtained through D,L-lactide ROP catalyzed by (LTi–O)_2_ were investigated.

## Materials and methods

### Materials

All of the experiments were carried out in dry and purified nitrogen (99.99%). D,L-lactide was synthesized in our laboratory (300 mL D,L-lactic acid and ZnO catalysts were added to 500ml three-mouth flask, and dehydrated for about 4 h at 120~140°C and ordinary pressure. Then increased the reaction temperature to 180~240°C, and injected nitrogen for 2 h. The crude product of D,L-lactide was obtained. Finally, the D,L-lactide was purified through crystallization with ethyl acetate, and dried in vacuum at 35°C for 48 h). Toluene dehydration was then performed with calcium hydride. Toluene, *n*-hexane, calcium hydride tetraethyl titanate, ethanediamine, ethanol, and other biochemical reagents of analytical grade were purchased from Chongqing Medicines Co., Ltd. (Chongqing, China). 3,5-Di-tertbutylsalicylidene was supplied by Galaxy Chemical Co., Ltd. (Wuhan, China). 1- to 2-day-old wistar rats were purchased from experimental animal center of Third Military Medical University.

### Preparation of (LTi–O)_2_

**Fig 1:**
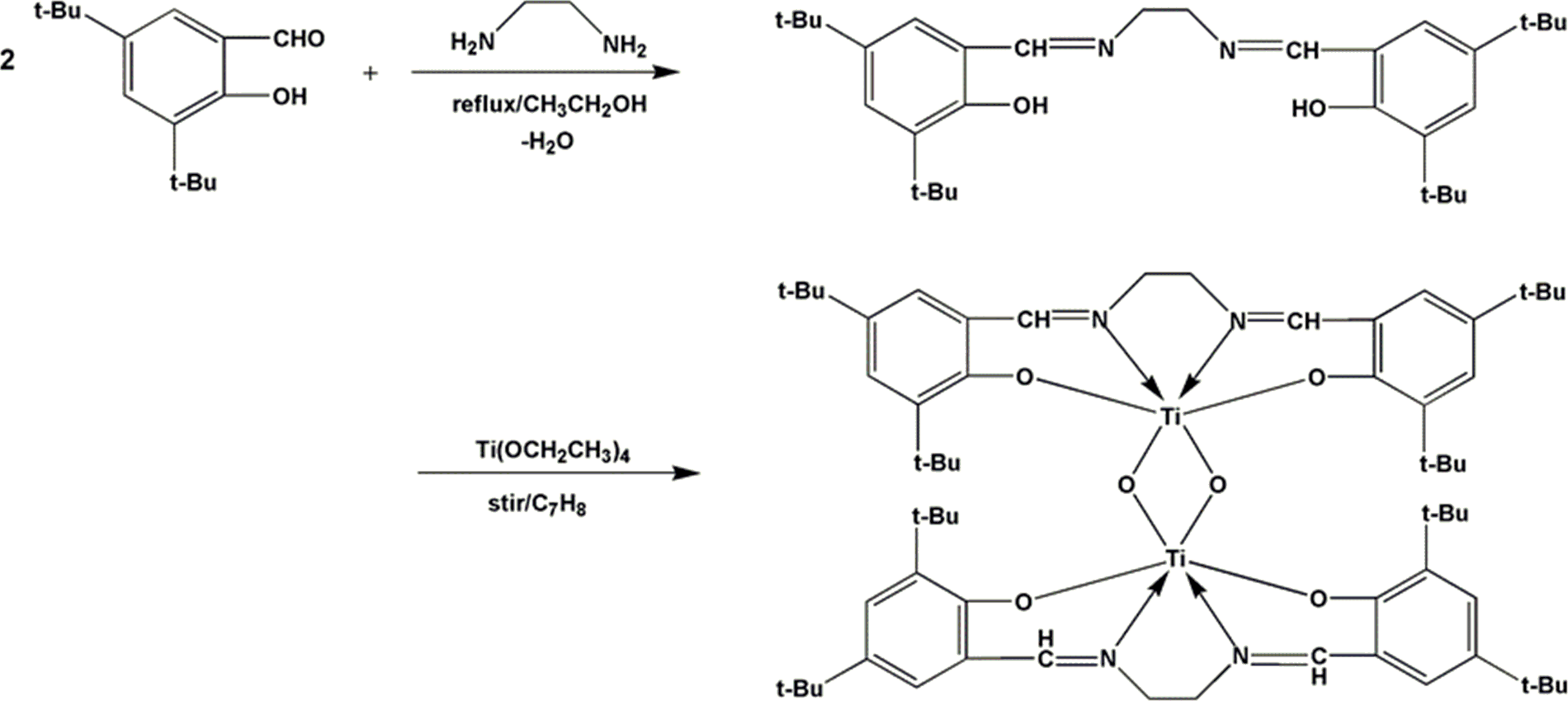
The synthetic route of (LTi-O)_2_.

The synthetic route of (LTi–O)_2_ is shown in Fig 1.

Preparation of salen ligand (L): 3,5-Di-tert-butylsalicylaldehyde (9.4 g, 0.04 mol) was dissolved in 60 ml of ethanol at about 80°C. After the aldehyde solution was stirred for 1 h, the ethylenediamine (1.2 ml, 0.02 mol) was added, and a yellow lamellar solid was quickly precipitated. The solid was filtered and washed with heat ethanol and then dried under vacuum for 24 h (yield, 79%). The following details were obtained: ^1^H NMR (S1 Fig) (CDCl_3_, 500 MHz, 25°C): δ = 1.338 (s, 18H, C (CH_3_)_3_), 1.493 (s, 18H, C (CH_3_)_3_), 3.951 (s, 4H, CH_2_), 7.118 (d, 2H, J = 1.2 Hz, Ar–H), 7.421 (d, 2H, J = 1.5 Hz, Ar–H), 8.431 (s, 2H, CH), 13.678 (s, 2H, OH). Analysis calculated for C_32_H_48_O_2_N_2_: C, 78.00; H, 9.82; N, 5.69. Found: C, 79.9; H, 9.80; N, 5.66.

Preparation of (LTi–O)_2_: (LTi–O)_2_ was prepared via an alcohol exchange method between tetraethyl titanate and the ligand at a 1:1 molar ratio in a toluene medium under dry and purified nitrogen. The mixture was stirred for 24 h at room temperature. The yellow solid was then crystallized from the toluene medium (yield, 65%). The following details were obtained: ^1^H NMR (S2 Fig) (CDCl_3_, 500 MHz, 25°C): 1.272 (s, 18H, C (CH_3_)_3_), 1.363 (s, 18H, C (CH_3_)_3_), 3.383 (d, 4H, J = 3.6 Hz, CH_2_), 4.418 (d, 4H, J = 3.3 Hz, CH_2_), 6.994 (d, 4H, J = 1.2 Hz, Ar-H), 7.395 (d, 4H, J = 1.2 Hz, Ar-H), 8.082 (s, 2H, CH). Analysis calculated for C_64_H_92_N_4_O_6_Ti_2_: C, 69.30; H, 8.36; N, 5.05. Found: C, 69.29; H, 8.34; N, 5.03.

### Characterization

FTIR spectra were recorded on a Perkin-Elmer GX spectrometer (Perkin-Elmer, USA) on KBr discs. ^1^H NMR and ^13^C NMR spectra were obtained with an Avance-500 NMR spectrometer (Bruker, Switzerland) in deuterated chloroform (CDCl_3_) by using tetramethylsilane as an internal standard. Elemental analysis was carried out on a Vario EL III elemental analysis instrument (Elementary, Germany) through ICP–atomic emission spectroscopy. TG/DSC analysis was performed on an STA449C instrument (Netzsch, Germany) under an argon atmosphere at a heating rate of 10°C min^−1^.

### Polymerization experiments

The bulk polymerizations of D,L-lactide were conducted in vacuum-sealed glass ampules after all glass ampules were dried at 180°C for 3 h in a muffle furnace. The glass ampules were charged with different molar ratios of D,L-lactide and (LTi–O)_2_, bubbled with pure N_2_ (99.99%) for 10 min to remove oxygen completely, quickly heat sealed, and immersed in a thermostatic oil bath at a setting temperature. After a predetermined time, the glass ampules were removed and quenched to around 25°C to stop the polymerization. Afterward, the polymers were purified by dissolving them in dichloromethane and precipitating from excessive amounts of cold methanol. PDLLA was dried in vacuum at 35°C for 24 h. Monomer conversion determinations were monitored in terms of the integration ratio of monomers to polymer methane or methyl resonances in ^1^H NMR (CDCl_3_). The molecular weight and PDI of PDLLA were determined by using a gel permeation chromatography instrument equipped with a multi-angle laser light scattering detector. Molecular weights were calibrated with polystyrene standards. Tetrahydrofuran was used as an eluent at a flow rate of 1.0 mL min^−1^ at 40°C.

### Analysis of physical/chemical properties of PDLLA

#### Preparation of PDLLA thin films

PDLLA dichloromethane solution (0.5 g·ml^−1^) was poured into a homemade PTFE mold covered with a homemade carton. The sample was dried at room temperature for 24 h and then dried in vacuum-drying equipment for 48 h. Then, the shaped sample was removed and dried in a vacuum-drying equipment at room temperature for about 1 week to eliminate methylene chloride completely. Finally, the resulting PDLLA film was cut into an appropriate shape depending on experimental requirements.

#### Determination of static contact angle of water

At room temperature, the slide fixed with the PDLLA film was secured on the glass stage of an instrument used to measure the contact angle. Then, the contour of a drop of distilled water, which was vertically dropped onto the PDLLA film surface with a 0.1 μl precision syringe, was observed under a microscope (magnification of 10×) on the instrument for contact angle measurement. Three static contact angles of water were measured on the surface of each sample, and the calculated averages were recorded as the static contact angles of water of the samples.

#### Determination of water uptake ratios

PDLLA film (0.3 g) was immersed in distilled water at 37°C for 24 h. Then, the film was taken out, and water on the surface was removed with a filter paper. Finally, the water uptake ratio of the sample could be calculated with the following equation:

$$water\;uptake\;ratio\;(\%)=\frac{W_{1}-W_{0}}{W_{0}}$$

where *W*_0_ and *W*_1_ are the weights of the PDLLA film before and after water absorption, respectively. The samples were tested in parallel three times, and the average was calculated for the following analysis.

1) In vitro degradation test

In this study, PBS at pH 7.4 and distilled water at pH 6.46 were used as hydrolysis media. PDLLA samples were immersed in the hydrolysis media and placed in an incubator at 35 ± 0.5°C. The samples were subjected to index determinations once a week. The weight loss (%) and water uptake ratios (%) could be calculated using the following equations:

$$Weight\;loss\;(\%)=\frac{m_{0}-m_{t}}{m_{0}}$$

$$Water\;uptake\;ratio\;(\%)=\frac{m_{wt}-m_{0}}{m_{0}}$$

where *m*_0_ and *m*_t_ are the dry weights of the sample film before and after the time *t* of degradation, and *m*_wt_ is the wet weight of the sample film after *t* of degradation.

### Analysis of PDLLA biocompatibility

#### Preparation of PDLLA thin films

PDLLA dichloromethane solution (50 mg·ml^−1^; 50 μl) was spread on circular cover glasses with Transferpettor. The solution was volatilized and vacuum dried at room temperature for 72 and 48 h, respectively. Each sample was composed of 20 parallel samples. Afterward, the circular PDLLA films were sterilized through UV irradiation for 0.5 h and then stored in a vacuum-drying oven for further tests.

#### Primary osteoblast culture

Firstly, Medical Ethics Committee of Southwest Hospital approved the study. Under sterile conditions, four 1- to 2-day-old wistar rats were anesthetized with 75% alcohol for 5 minutes, and then the cranium of wistar rats was harvested and washed with PBS to clear the periosteum and cartilaginous tissues. The cranium was cut into 0.5 mm^3^ fragments and cultured using a primary culture technique with mature osteoblasts.

#### Cell morphological observation

A 24-hole plate arranged with sample cover glasses was initially sterilized through UV irradiation for 24 h on a superclean bench. The osteoblasts were seeded at a density of 1×10^4^ per hole, and 2 ml of DMF12 was added. The cells were cultured in an incubator at a constant temperature of 37°C. The morphological characteristics of the cells were observed under an inverted phase-contrast microscope on day 3 of culture.

#### Determination of cell increment

In this study, the MTT method was used to evaluate the increment in osteoblasts on the PDLLA material. The osteoblasts were seeded into a 24-hole plate arranged with sample cover glasses at a density of 1×10^4^ per hole. DMF12 (2 ml) was subsequently added, and the cells were cultured in an incubator at a constant temperature of 37°C. Cell proliferation was evaluated with MTT assay 1, 3, 5, and 7 days after inoculation.

Furthermore, the primary osteoblast culture experiment was approved by the Medical Ethics Committee of Southwest Hospital.

## Results and discussion

### Characterization of (LTi–O)_2_

**Fig 2:**
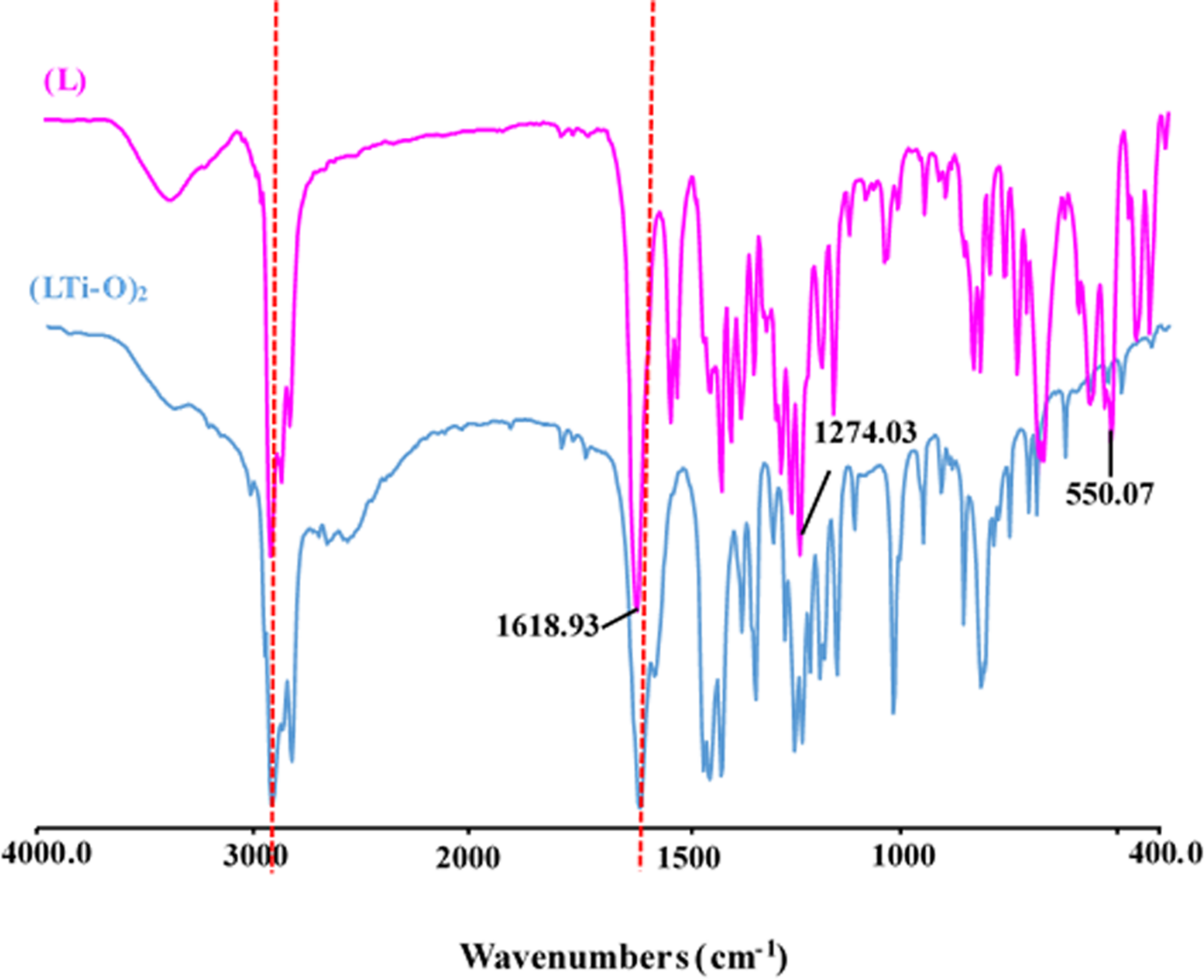
FTIR spectra of L and (LTi-O)_2_.

FTIR spectra: The FTIR spectra of the prepared salen ligand (L) and the (LTi–O)_2_ metal complex are shown in Fig 2. The spectrum of (LTi–O)_2_ was similar to that of L. The absorption peaks at 1618.93 and 1274.03 cm^−1^ were attributed to imine (–CH = N–) and stretching vibration of phenolic hydroxyl (Ar–O), respectively. The red shift of the corresponding absorption peaks in the spectra of (LTi–O)_2_ and the newly emerged peak at 550.07 cm^−1^ indicated the successful coordination between a N atom in L and a Ti atom [16, 17]. Therefore, the FTIR spectra confirmed that (LTi–O)_2_ was synthesized successfully.

**Fig 3:**
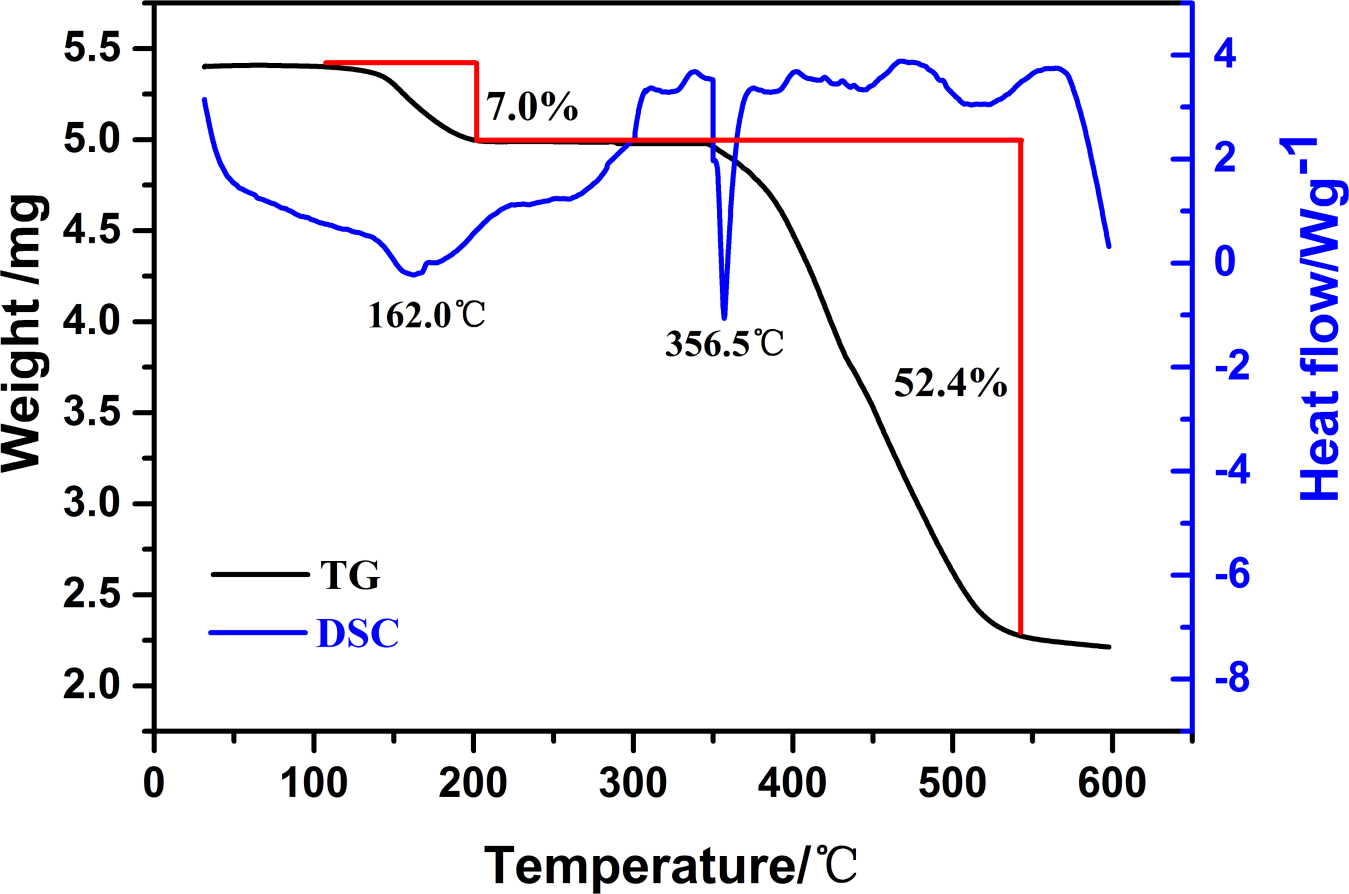
TG/DSC analysis of (LTi-O)_2_.

TG/DSC: The thermal stability of (LTi–O)_2_ was assessed through TG/DSC analysis (Fig 3). Two obvious weight loss stages were observed in the thermal weight curve. The first stage (113.0°C–201.5°C, 7.0%) was considered for the evaporation of toluene, which was confirmed to exist in (LTi–O)_2_. The second stage (348.5°C–543.7°C, 52.4%) was accounted for the final decomposition of (LTi–O)_2_. The two endothermic peaks in the DSC curve formed at 162°C and 356.5°C, respectively. No weight loss of (LTi–O)_2_ occurred at temperatures lower than 348.5°C, indicating that (LTi–O)_2_ was suitable for the bulk polymerization of D,L-LA in this temperature range.

### ROP of D,L-LA by (LTi–O)_2_

#### ROP characteristics

**Fig 4:**
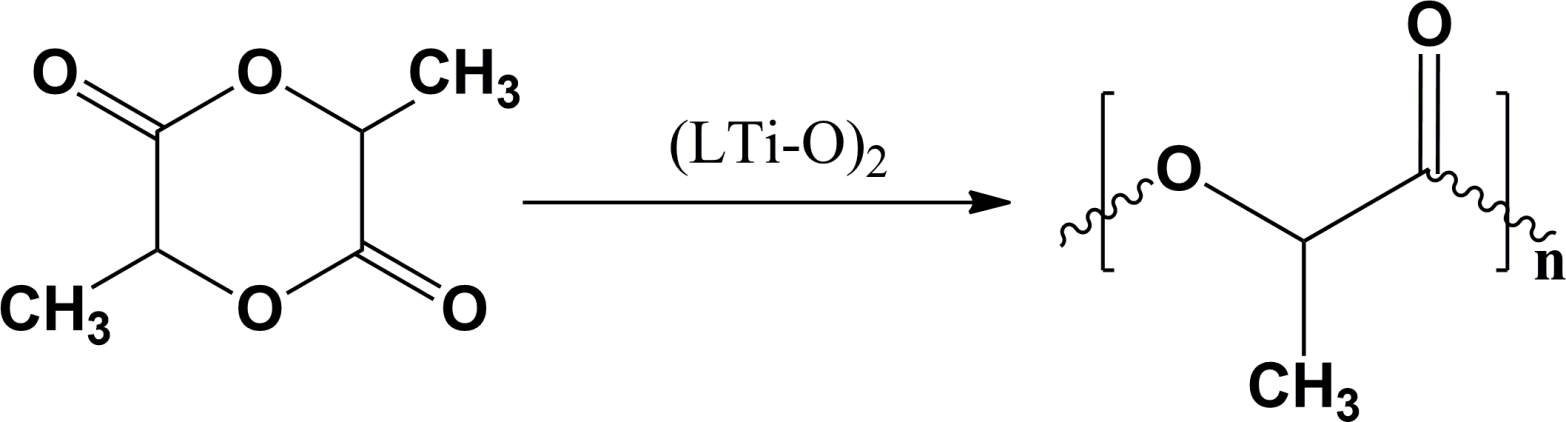
ROP of D, L-LA employing (LTi-O)_2_ as catalyst.

**Fig 5:**
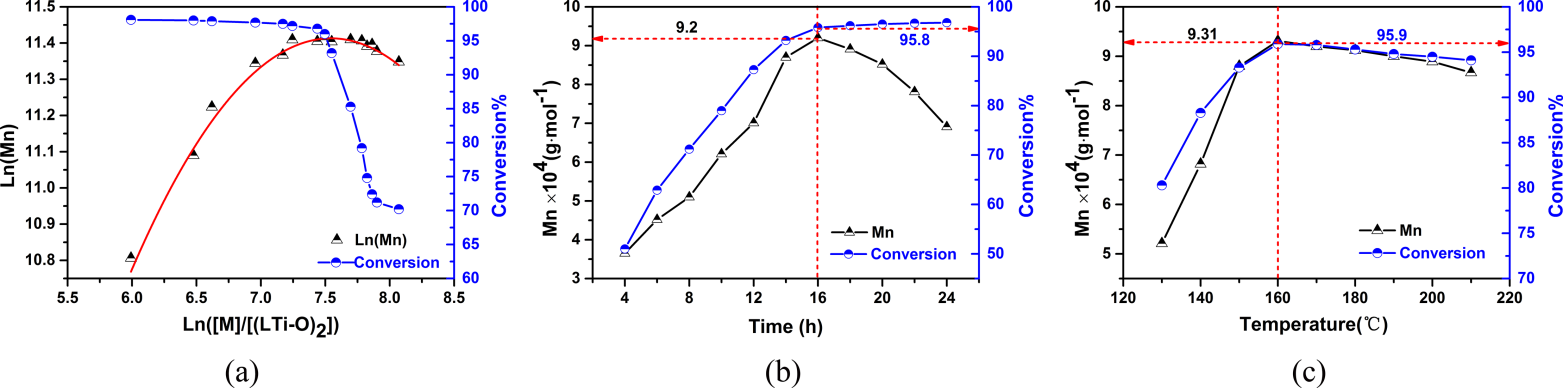
Effect of molar ratio of monomer (D, L-LA) and catalyst ((LTi-O)_2_) (a), reaction time (b) and polymerization temperature (c) on ROP of D, L-LA.

D,L-LA ROP involving (LTi–O)_2_ as a catalyst (Fig 4) under solvent-free conditions was systematically examined (S1 Table). The obtained PDLLA was also characterized through ^1^H NMR and ^13^C NMR spectroscopy (S3 Fig and S4 Fig). Pre-experimental results showed that the molar ratio of the monomer (D,L-LA) and the catalyst [(LTi–O)_2_] ([M]/[(LTi–O)_2_]), polymerization temperature, and reaction time influenced polymerization. Therefore, the effects of these factors on the number–average molecular weight (*M*_n_) and monomer conversion ratio were investigated (Fig 5) (Five experiments were carried out in parallel with each condition, and the average deviation is 0.26).

Fig 5(A) illustrates the effect of the molar ratio of D,L-LA and (LTi–O)_2_ on *M*_n_ of PDLLA and the monomer conversion ratio (conditions: 160°C, 16 h). The red solid line represents the nonlinear fit of the second-order quadratic model for the experimental data: *Y* = -0.256*X*^2^+3.8617*X*-3.1464, R^2^ = 0.9924 (for M_n_). *M*_n_ of PDLLA initially increased to a maximum value of 9.09×10^4^ g·mol^−1^ and then slightly decreased as the molar ratio increased. However, the monomer conversion ratio gradually declined as the molar ratio increased. During ROP, the number of catalytic active sites depends on the initial catalyst concentration. An insufficient number of catalytic active sites due to low initial catalyst concentration create difficulty in initiating the ROP of D,L-LA and maintaining the chain growth of a polymer. An excessive number of active sites is also detrimental. Although excessive active sites can increase the number of catalytic active sites of a monomer and lead to an increased conversion rate, a polymer intermolecular or intramolecular ester exchange reaction occurs, thereby decreasing *M*_n_. This trend is consistent with lactide ROP catalyzed by tin octoate or an alkyl metal compound.

Fig 5(B) and 5(C) illustrate the effects of reaction time (conditions: [M]/[(LTi–O)_2_] = 1800:1, 160°C) and temperature (conditions: [M]/[(LTi–O)_2_]: 1800:1, 16 h) on *M*_n_ of PDLLA and monomer conversion ratio, respectively. After 16 h of polymerization, the reaction time was further prolonged, the monomer conversion ratio increased very slowly, and *M*_n_ of PDLLA declined. This phenomenon could be explained as follows. 1) After a long period of polymerization, most of the monomers were consumed, and a low monomer concentration was consequently retained. Therefore, the reaction rate decreased, and the increased rate of conversion ratio declined. 2) After polymerization was performed for an optimum duration of 16 h, the occurrence of side reactions, such as an unbalanced insertion of lactide into a polymer chain and a polymer intermolecular or intramolecular ester exchange reaction, increased, resulting in a decrease in *M*_n_. D,L-LA ROP catalyzed by (LTi–O)_2_ was conducted at 130°C–210°C based on the melting point (126°C) of lactide and the decomposition temperature (348.5°C, obtained by TG/DSC analysis) of (LTi–O)_2_ [Fig 5(C)]. *M*_n_ of PDLLA and the monomer conversion ratio initially increased to the maximum level (9.31×10^4^ mol·L^−1^ and 95.9%) at 160°C and then declined. This decrease could be attributed to the partial decomposition of PDLLA into lactide or an oligomer.

The PDIs of the obtained PDLLA (S1 Table) were all below 1.22, which was lower than that of PLA obtained through traditional synthesis [18, 19]. Low PDIs indicated the high controllability of *M*_n_ in the ROP of D,L-LA, and such controllability could be attributed to the large steric hindrance of (LTi–O)_2_, thereby preventing the occurrence of side reactions, such as polymer intermolecular or intramolecular ester exchange reaction.

#### Reaction kinetics and mechanism

Kinetic studies were further conducted under different reaction conditions to establish the reaction order of the monomer and (LTi–O)_2_ in the ROP of D,L-LA. The kinetic equation could be described as −*d*[M]/*dt* = *k*[M]^*m*^[(LTi–O)_2_]^*n*^, where [M] and [LTi–O)_2_] are the respective concentrations of the monomer and (LTi–O)_2_, *m* and *n* are the reaction orders, and *k* is the rate constant.

**Fig 6:**
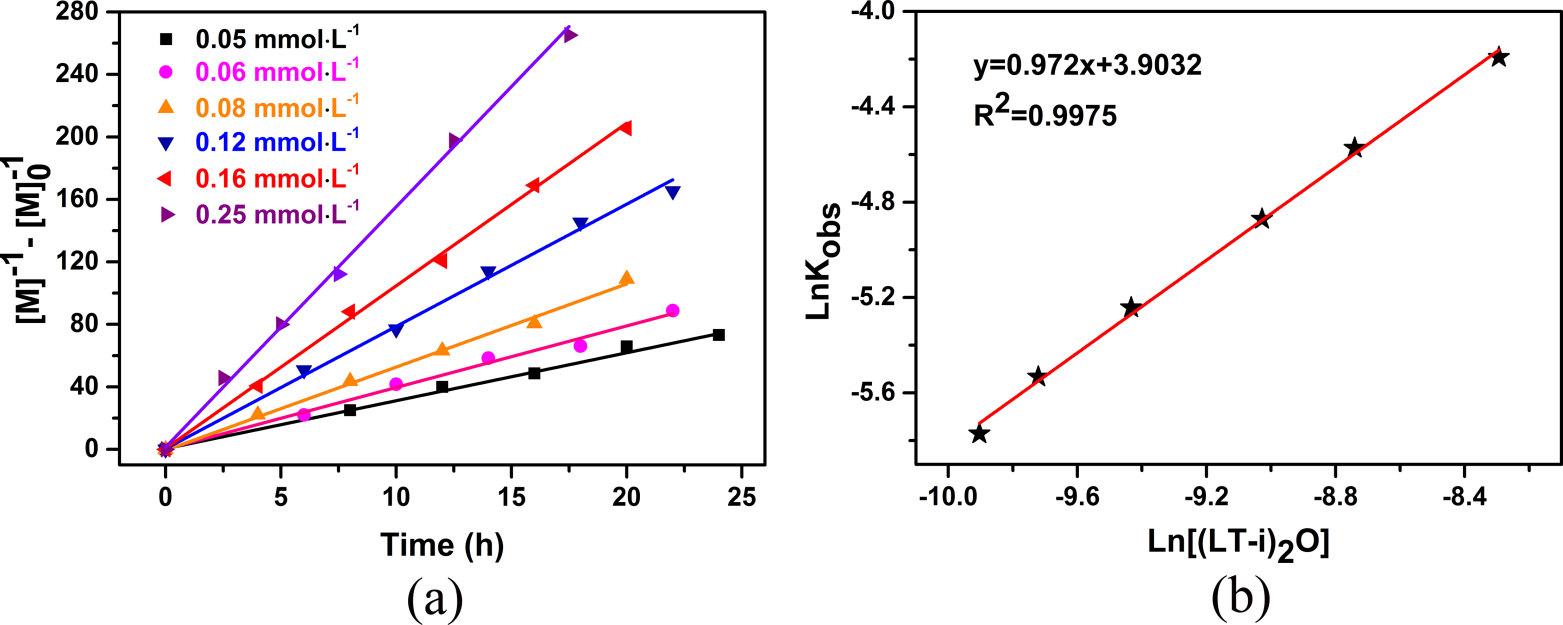
ROP kinetics: Second-order kinetic plots for D, L-LA polymerization vs. time with different catalyst concentrations at 160°C and [M_0_] = 0.1mol·L^-1^(a); Double-logarithmic line fitting of k_obs_ vs. [(LTi-O)_2_] with different catalyst concentrations at 160°C and [M_0_] = 0.1mol·L^-1^(b).

To determine *m* of [M], we expressed the kinetic equation as follows: −*d*[M]/*dt* = *k*_obs_[M]^*m*^, where *k*_obs_ = *k*[(LTi–O)_2_]^*n*^). The (1/[M]–1/[M]_0_) versus time plots exhibited a linear variation in each (LTi–O)_2_ concentration at 160°C [Fig 6(A)] and indicated the second order of the monomer concentration. Thus, the kinetic equation could be expressed as follows:–*d*[M]/*dt* = *k*_obs_[M]^2^.

The reaction order *n* of [(LTi–O)_2_] and the rate constant *k* (conducted at 160°C) could be obtained from the logarithmic linear fit of *k*_obs_ with [(LTi–O)_2_] [Fig 6(B)]. *n* of [(LTi–O)_2_] was 0.972, which was obtained from the slope of the regression line. This value was close to 1, indicating that the polymerization of D,L-LA was the first order of the (LTi–O)_2_ concentration. Therefore, the kinetic equation of D,L-LA ROP catalyzed by (LTi–O)_2_ could be expressed as–*d*[M]/*dt* = *k*[M]^2^[(LTi–O)_2_]^1^, which was the same as that of the reported metal complex catalyst system but different from that of a typical lactide ROP (–*d*[M]/*dt* = *k*[M]^1^[catalyst]^1^) [20, 21]. Such kinetic orders obtained in this work were attributed to the aggregation of a metal catalyst or growing polymer chains, implying that one (LTi–O)_2_ molecule and two monomers were involved in the transition state of the propagation event.

In addition to *n*, *ln k* was determined from the logarithmic linear regression of *k*_obs_ and [(LTi–O)_2_] and was considered the Y axis intercept equivalent to 3.9032. Therefore, the apparent *k* at 160°C was 49.56 mol^−2^·L^2^·h^−1^. Similarly, *k*_obs_ and the logarithmic linear regression equation of *k*_obs_ with [(LTi–O)_2_] at different reaction temperatures are listed in Table 1. Our results yielded apparent *k* of 14.00, 22.26, 87.70, and 152.52 mol^−2^·L^2^·h^−1^ at 140°C, 150°C, 170°C, and 180°C, respectively.

**Table 1:** The k_obs_ and equation of logarithm linear regression of k_obs_ with [(LTi-O)_2_] at different reaction temperature.

k_obs_ / mol^-1^·h^-1^							Eq. of linear regression (Ln(kobs) vs. Ln[(LTi-O)_2_])
T/°C	[(LTi-O)_2_]/ m mol·L^-1^						
	0.05	0.06	0.08	0.12	0.16	0.25	
140	0.76	0.91	1.20	1.80	2.40	3.73	y = 0.9923x+2.6390, R^2^ = 0.9923
150	1.26	1.51	2.01	3.00	3.98	6.18	y = 0.9873x+3.1026, R^2^ = 0.9947
160	3.26	3.89	5.14	7.63	10.09	15.58	y = 0.9724x+3.9032, R^2^ = 0.9975
170	4.38	5.25	7.00	10.51	14.01	21.89	y = 1.0002x+4.4739, R^2^ = 0.9962
180	7.54	9.05	12.06	18.11	24.15	37.75	y = 1.0012x+5.0273, R^2^ = 0.9987

**Fig 7:**
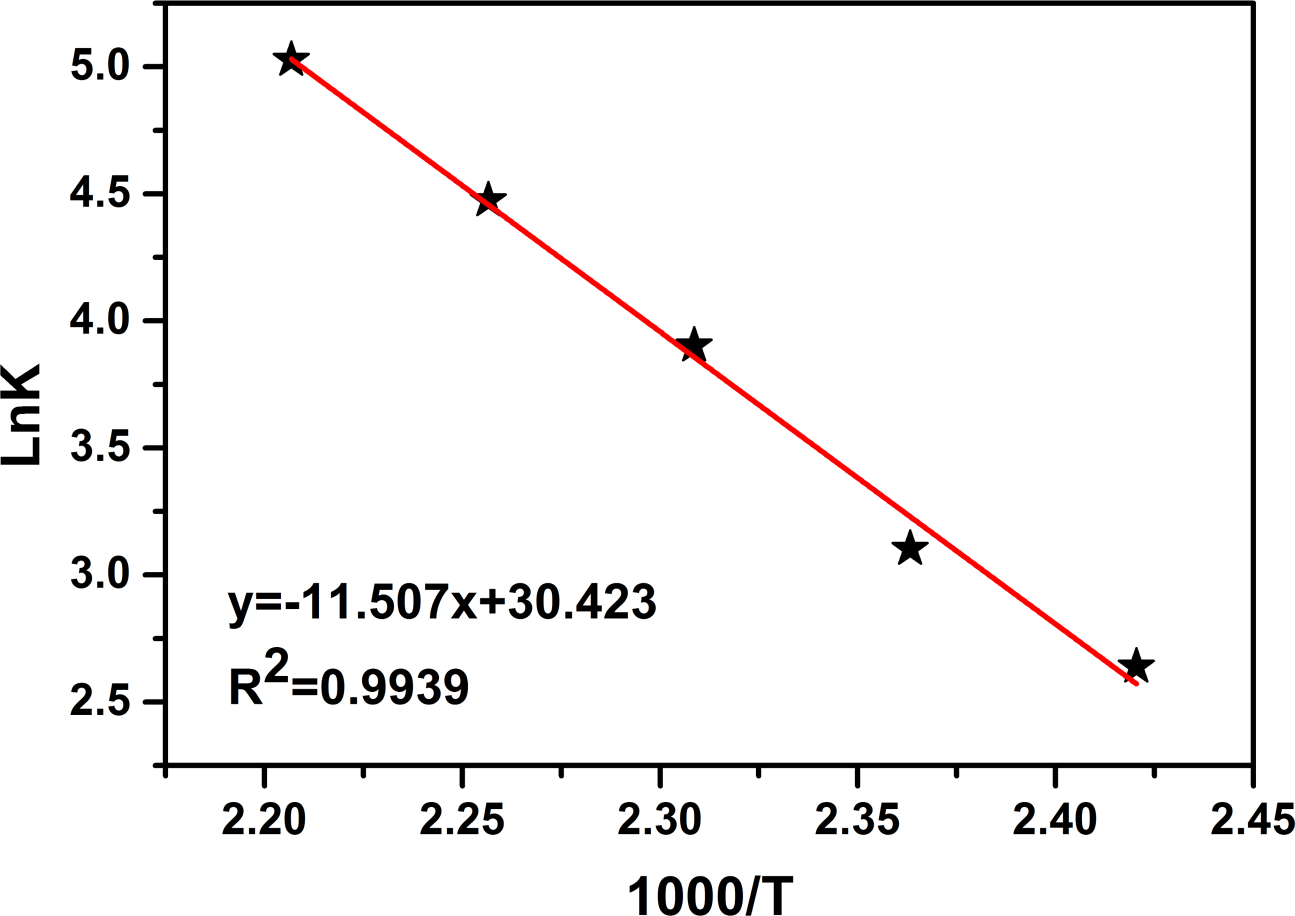
Semi-logarithmic line fitting of k vs. 1000/T ([M]_0_ = 0.1 mol· L^−1^).

According to Arrhenius equation (*ln k* = *ln A*−*E*_a_/*RT*), the reaction activation energy *E*_a_ (95.67 kJ·mol^−1^) and the frequency factor *A* (1.63×10^13^) of D,L-LA ROP catalyzed by (LTi–O)_2_ were obtained from the linear fitting of *ln k* versus 1000/*T* (Fig 7). The obtained *E*_a_ was within the scope of the reported lactide ROP catalyzed by the metal complex, thereby verifying the reliability of the experimental data in this work [22, 23].

### Performance evaluation of PDLLA

#### Physical and chemical property of PDLLA

Biodegradable PLA polymer materials used in tissue engineering should possess the corresponding degradation and absorption rates with the growth rate of cells or tissues in vivo [24–26]. Therefore, studies on the physical and chemical properties of PLA polymer materials and their degradation performance may provide a basis for their preparation, processing, and application. The degradation of biodegradable polymer materials in organisms elicits physical and chemical effects. The hydrophilic/hydrophobic properties of materials are among the main factors affecting material degradation. Therefore, the hydrophilicity and hydrophobicity of materials should be investigated. In our study, PDLLA-1 (*M*_n_ = 9.20×10^4^ mol·L^−1^, PDI = 1.13) was selected as the evaluating polymer material. For comparison, PDLLA-2 (*M*_n_ = 9.01×10^4^ mol·L^−1^, PDI = 1.33) obtained through D,L-LA ROP catalyzed by stannous octoate was chosen as a polymer material.

The static contact angle of water and 24 h water uptake ratios of these two polymers are listed in Table 2. The static contact angle of water of PDLLA-2 was lower and its 24 h water uptake ratios were higher than those of PDLLA-1, indicating that the hydrophilicity of the former was higher than that of the latter. *M*_n_ and PDI of PDLLA-2 were higher than those of PDLLA-1, implying that the hydrophilicity of PDLLA-2 was enhanced by numerous oligomers and high concentrations of hydrophilic carboxyl group. The results on the 24 h water uptake ratios confirmed that the hydrophilicity of PDLLA-2 was higher than that of PDLLA-1.

**Table 2:** Static water contact angle and 24h water-uptake ratios of polymers.

Samples^a^	Static water contact angle^b^	24h water-uptake ratios^b^(%)
PDLLA-1	100.0±0.01	1.42±0.32
PDLLA-2	83.8±0.02	2.03±0.26

^a^ PDLLA-1 (Mn = 9.20×104 mol· L-1, PDI = 1.13); PDLLA-2 (Mn = 9.01×104 mol· L-1, PDI = 1.33)

^b^ All the experiments were conducted in 3 parallel experiments, and the average values were calculated and used as the result for the subsequent analysis.

**Fig 8:**
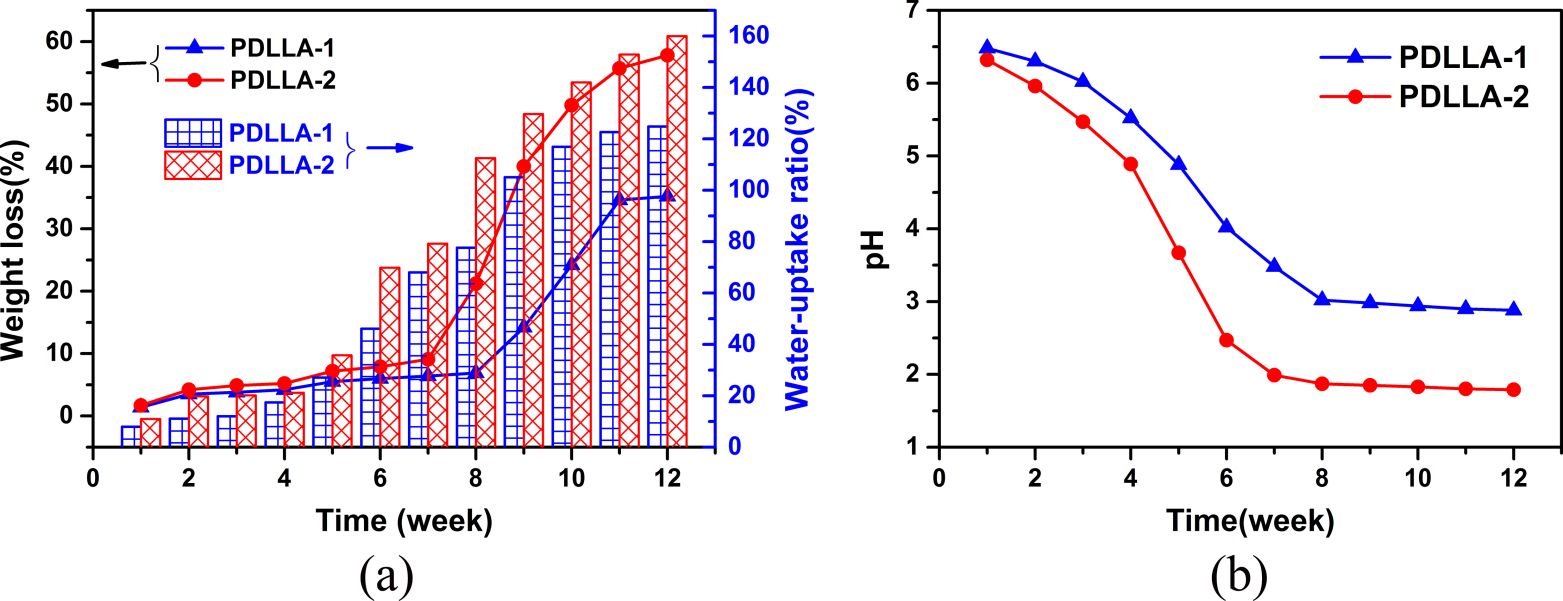
Degradation property of polymers: the change of weight loss (a), water-uptake ratios (a) and pH (b) with time.

The degradation properties of polymers and the evaluating indicators, namely, weight loss (%), water uptake ratios (%), and solution pH, are illustrated in Fig 8 (three parallel experiments were carried out under each condition, and the average deviation was 0.5%). Variations in the indicators of PDLLA-1 were similar to those of PDLLA-2, suggesting that the two polymers underwent similar degradation processes. However, PDLLA-2 was degraded more rapidly than PDLLA-1 in each degradation time point, and the former entered the rapid degradation stage earlier than the latter did, that is, PDLLA-2 was rapidly degraded on week 8, whereas PDLLA-1 was quickly degraded on week 9 [Fig 8(A)]. The loss of polymer weight indicated that the polymer material was absorbed by the medium. Similarly, the higher PDI of PDLLA-2 than that of PDLLA-1 implied that the high oligomer content and hydrophilic carboxyl group concentration accelerated the degradation rate and thus enhanced PDLLA-2 degradation. The high concentration of the hydrophilic carboxyl group could also improve the hydrophilicity and water absorption of PDLLA-2 [Fig 8(A)]. Changes in solution pH with degradation time for PDLLA-1 and PDLLA-2 could be divided into three stages: slow decline, weeks 1–3; quick decline, weeks 4–7; and slight decline, weeks 8–12 [Fig 8(B)]. However, the pH decline rate of PDLLA-1 was slower than that of PDLLA-2 in each degradation time, and this phenomenon was consistent with the variation pattern of weight loss and water uptake ratios. Implant materials in tissue engineering must be processed for a certain stable period, which is at least 8 weeks, to favor the growth of organisms and influence loaded drugs. Therefore, PDLLA-1 that entered the rapid degradation stage on week 9 was more suitable for tissue engineering than PDLLA-2.

#### Biocompatibility

**Fig 9:**
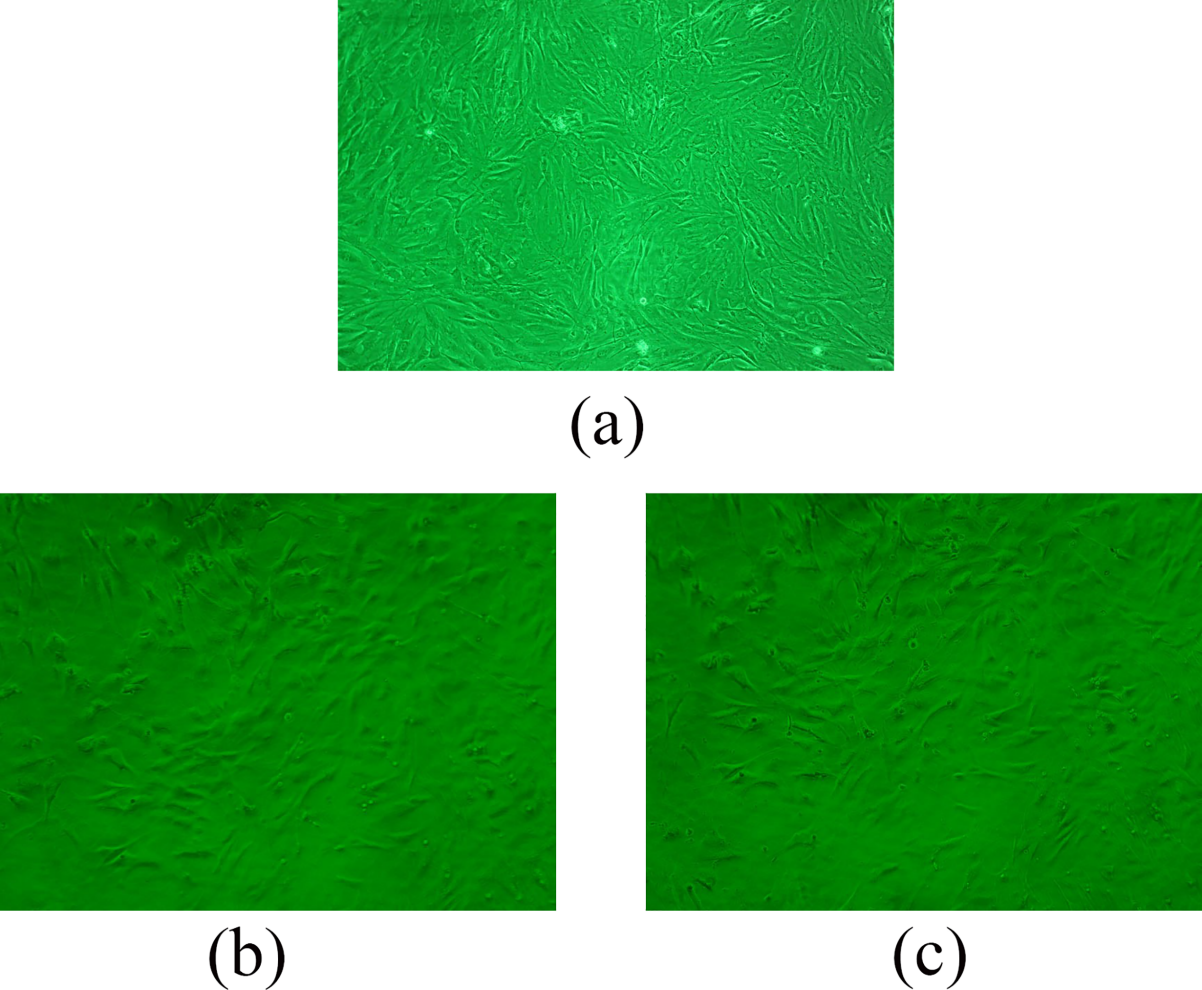
Cell morphology at 3^th^ day of culture: on glass(a); on PDLLA-1 (b); on PDLLA-2(c).

Medical materials implanted in the human body must exhibit good biocompatibility. Good biocompatibility involves two aspects, namely, biological safety principle, which represents toxic/side effects on the human body, and biological function principle, which includes the acceptability of implanted materials in the human body. In vitro cell culturing is the main method used to evaluate the biocompatibility of implant materials. In vitro biocompatibility test, which is simple and repeatable, has been the preferred method to assess the biocompatibility of materials [27, 28]. Direct contact method provides the advantage of high susceptibility to toxicity testing for the evaluation of the biocompatibility of PDLLA-1 and PDLLA-2. In this method, cells are seeded directly onto the surface of a material and cultured for several days. In our study, the cells spreading on the surface of the materials on day 3 of culture are illustrated in Fig 9.

**Fig 10:**
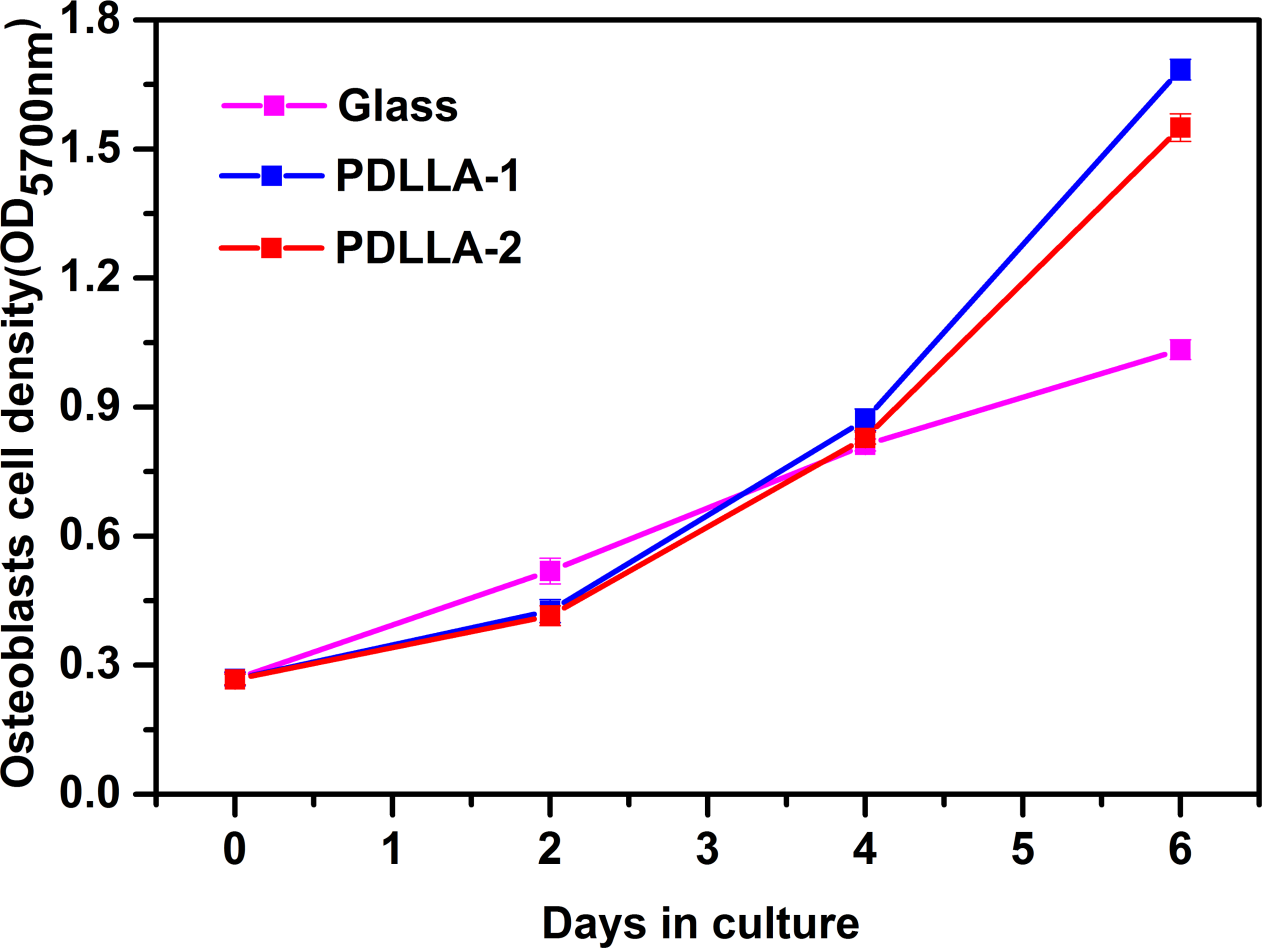
Growth curves of osteoblasts cells on materials.

The osteoblasts were well distributed on the surface of the materials. Most of the cells resembled short spindles, whereas the other cells were circular and triangular. This observation was consistent with the reported morphological characteristics of osteoblasts. In terms of cell density and distribution, the morphological characteristics of the cells on the two kinds of materials were almost the same, and all preceded the blank sample [Fig (9A)]. No deformed or dead cells were observed in the experimental group, indicating that the materials did not affect cell viability. The osteoblast multiplication on the test materials is presented in Fig 10. With the extension of culture time, the number of osteoblasts in each group increased, and the cells on PDLLA-1 showed the strongest multiplication vitality after 2 days of culture. The higher concentration of hydrophilic carboxyl group in PDLLA-2 than in PDLLA-1 partly inhibited cell growth and further affected cell proliferation.

To further evaluate cell proliferation on materials, we calculated the relative average proliferation rates of the cells at different time points (Table 3). On the first 2 days of culture, the glass sample showed the optimum cell growth. The hydrophobic group on the material was not conducive to cell adhesion. Thus, the cells underwent an adaptive process to grow on the material surface. Therefore, on the first few days of culture, most of the cells on PDLLA remained in the G_0_ phase. The cells on the surface of PDLLA gradually adapted to the external environment after they initially adjusted. Therefore, the cells began to divide and proliferated on days 3 to 4, and their proliferation rate increased rapidly. After 4 days of culture, the materials were covered with cells, and an excessive cell density inhibited further osteoblast proliferation, which was indicated as the decline in the relative average proliferation rate on days 5 to 7. Our analysis revealed that the obtained PDLLA-1 achieved a relatively excellent biocompatibility suitable for osteoblast culture.

**Table 3:** The relative average proliferation rate of cells in different time periods.

Relative average proliferation rate(%)			
Materials	Culture days		
	0→2	2→4	4→6
Glass	46.8	28.2	13.7
PDLLA-1	29.5	52.2	46.7
PDLLA-2	27.7	49.8	43.6

## Conclusions

A novel (LTi–O)_2_ metal complex was synthesized and fully characterized by a series of instrumental analytic methods. ^1^H NMR and FTIR indicated the presence of metal titanium atoms with N atoms in the ligand. TG/DSC analysis revealed that the (LTi–O)_2_ complex with a decomposition temperature of 348.5°C was suitable for the bulk polymerization of D,L-LA at the same temperature. The ROP of D,L-LA by (LTi–O)_2_ demonstrated that the molar ratio of the monomer (D,L-LA) and the catalyst, the polymerization temperature, and the reaction time influenced polymerization. PDLLA, with *M*_n_ of 9.31×10^4^ mol·L^−1^ and PDI of 1.13, could be obtained under the following conditions: [M]_0_/[(LTi–O)_2_I]_0_ = 1800, reaction time of 16 h, and reaction temperature of 160°C. The kinetic equation of the D,L-LA ROP catalyzed by (LTi–O)_2_ could be expressed as −*d*[M]/*dt* = *k*[M]^2^[(LTi–O)_2_]^1^, and *E*_a_ was 95.67 kJ·mol^−1^. PDLLA obtained through the D,L-LA ROP catalyzed by (LTi–O)_2_ exhibited a corresponding excellent degradation performance and biocompatibility. Therefore, the material is suitable for osteoblast culture and tissue engineering. In conclusion, the obtained novel (LTi–O)_2_ metal complex with a high catalytic activity and thermal stability could be employed as a catalyst for the synthesis of PLAs with low PDIs and toxicity and for tissue engineering applications.

## Supporting information

S1 Table ROP of D, L-LA catalyzed by (LTi-O)2 in the bulk phase. (DOCX)

S1 Fig ^1^H NMR spectra of L. (TIF)

S2 Fig ^1^H NMR spectra of (LTi-O)_2_. (TIF)

S3 Fig ^1^H NMR spectra of PDLLA. (TIF)

S4 Fig ^13^C NMR spectra of PDLLA. (TIF)

## Abbreviations

ROP: Ring-opening polymerization
FTIR: Fourier transform-infrared spectroscopy
^1^H NMR: ^1^H nuclear magnetic resonance
TG/DSC: Thermogravimetric/ differential scanning calorimetry
EA: Elemental analysis
PDLLA: Poly(D,L-lactide)
Mn: Number-average molecular weight
PDI: Polydispersity index
[M]: Monomer concentration
[(LTi-O)2]: (LTi-O)_2_ concentration
Ea: Reaction activation energy
Ti: Titanium
PLA: Polylactic acid
GPC: Gel permeation chromatography
