# Supplementary material for: Synthesis and characterization of salen-Ti(IV) complex and application in the controllable polymerization of D, L-lactide
Source: PLoS One. 2018 Aug 2;13(8):e0201054. doi: 10.1371/journal.pone.0201054 (PMC6071980; doi:10.1371/journal.pone.0201054)
Supplement: S1 Table — (DOCX) [file pone.0201054.s001.docx]

**S1 Table**

| Entry | [M]_0_/[(LTi-O)_2_I]_0_ | t(h) | T(℃) | PDI | M_n_^a^(×10^4^) | Conv (%) |
| --- | --- | --- | --- | --- | --- | --- |
| 1 | 400 | 16 | 160 | 1.21 | 4.93 | 98.1 |
| 2 | 650 | 16 | 160 | 1.16 | 6.55 | 98.0 |
| 3 | 750 | 16 | 160 | 1.14 | 7.48 | 97.9 |
| 4 | 1050 | 16 | 160 | 1.09 | 8.44 | 97.7 |
| 5 | 1300 | 16 | 160 | 1.12 | 8.64 | 97.5 |
| 6 | 1400 | 16 | 160 | 1.15 | 9.01 | 97.2 |
| 7 | 1700 | 16 | 160 | 1.16 | 8.97 | 96.8 |
| 8 | 1800 | 16 | 160 | 1.14 | 9.09 | 96.0 |
| 9 | 1900 | 16 | 160 | 1.13 | 8.96 | 93.2 |
| 10 | 2200 | 16 | 160 | 1.16 | 9.02 | 85.3 |
| 11 | 2400 | 16 | 160 | 1.20 | 8.99 | 79.2 |
| 12 | 2500 | 16 | 160 | 1.21 | 8.85 | 74.8 |
| 13 | 2600 | 16 | 160 | 1.22 | 8.89 | 72.4 |
| 14 | 2700 | 16 | 160 | 1.17 | 8.73 | 71.2 |
| 15 | 3200 | 16 | 160 | 1.20 | 8.48 | 70.2 |
| 16 | 1800 | 4 | 160 | 1.09 | 3.65 | 51.0 |
| 17 | 1800 | 6 | 160 | 1.15 | 4.52 | 65.9 |
| 18 | 1800 | 8 | 160 | 1.20 | 5.10 | 71.2 |
| 19 | 1800 | 10 | 160 | 1.11 | 6.21 | 79.0 |
| 20 | 1800 | 12 | 160 | 1.03 | 7.01 | 87.3 |
| 21 | 1800 | 14 | 160 | 1.16 | 8.70 | 93.2 |
| 22  2 | 1800 | 16 | 160 | 1.13 | 9.20 | 95.8 |
| 23 | 1800 | 18 | 160 | 1.19 | 8.91 | 96.2 |
| 24 | 1800 | 20 | 160 | 1.02 | 8.52 | 96.5 |
| 25 | 1800 | 22 | 160 | 1.15 | 7.81 | 96.7 |
| 26 | 1800 | 24 | 160 | 1.13 | 6.91 | 96.8 |
| 27 | 1800 | 16 | 130 | 1.03 | 5.21 | 75.2 |
| 28 | 1800 | 16 | 140 | 1.16 | 6.82 | 81.4 |
| 29 | 1800 | 16 | 150 | 1.19 | 8.17 | 88.1 |
| 30 | 1800 | 16 | 160 | 1.13 | 9.31 | 95.9 |
| 31 | 1800 | 16 | 170 | 1.16 | 9.20 | 94.2 |
| 32 | 1800 | 16 | 180 | 1.17 | 9.12 | 93.3 |
| 33 | 1800 | 16 | 190 | 1.02 | 9.00 | 92.8 |
| 34 | 1800 | 16 | 200 | 1.14 | 8.89 | 92.0 |
| 35 | 1800 | 16 | 210 | 1.16 | 8.67 | 91.1 |

^a^Determined from GPC instrument (THF at 1 ml per minute, relative to polystyrene standards).
