# Supplementary figures and images for: Synthesis and characterization of salen-Ti(IV) complex and application in the controllable polymerization of D, L-lactide
Source: PLoS One. 2018 Aug 2;13(8):e0201054. doi: 10.1371/journal.pone.0201054 (PMC6071980; doi:10.1371/journal.pone.0201054)

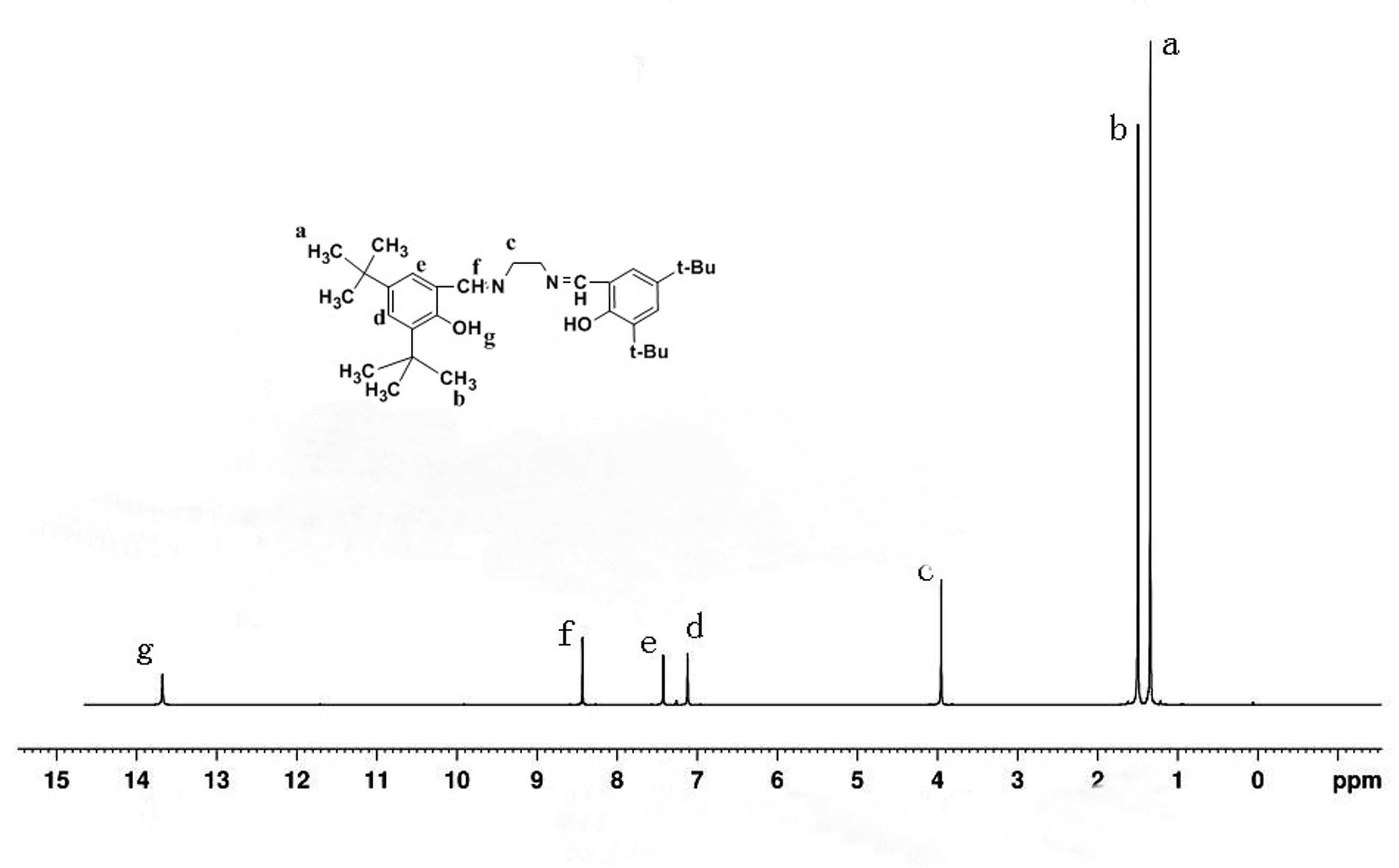

Supplement: S1 Fig — (TIF) [file pone.0201054.s002.tif]

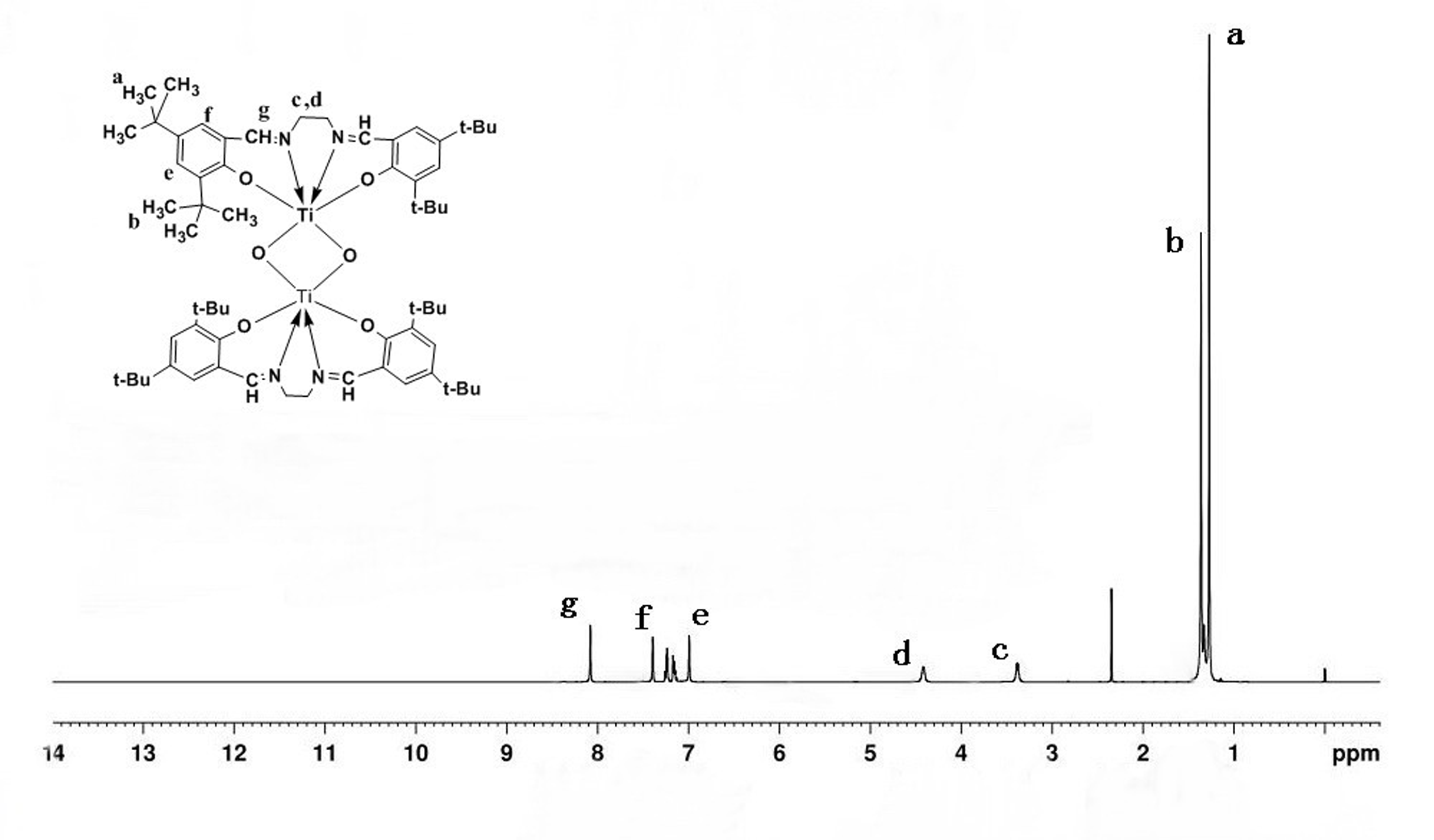

Supplement: S2 Fig — (TIF) [file pone.0201054.s003.tif]

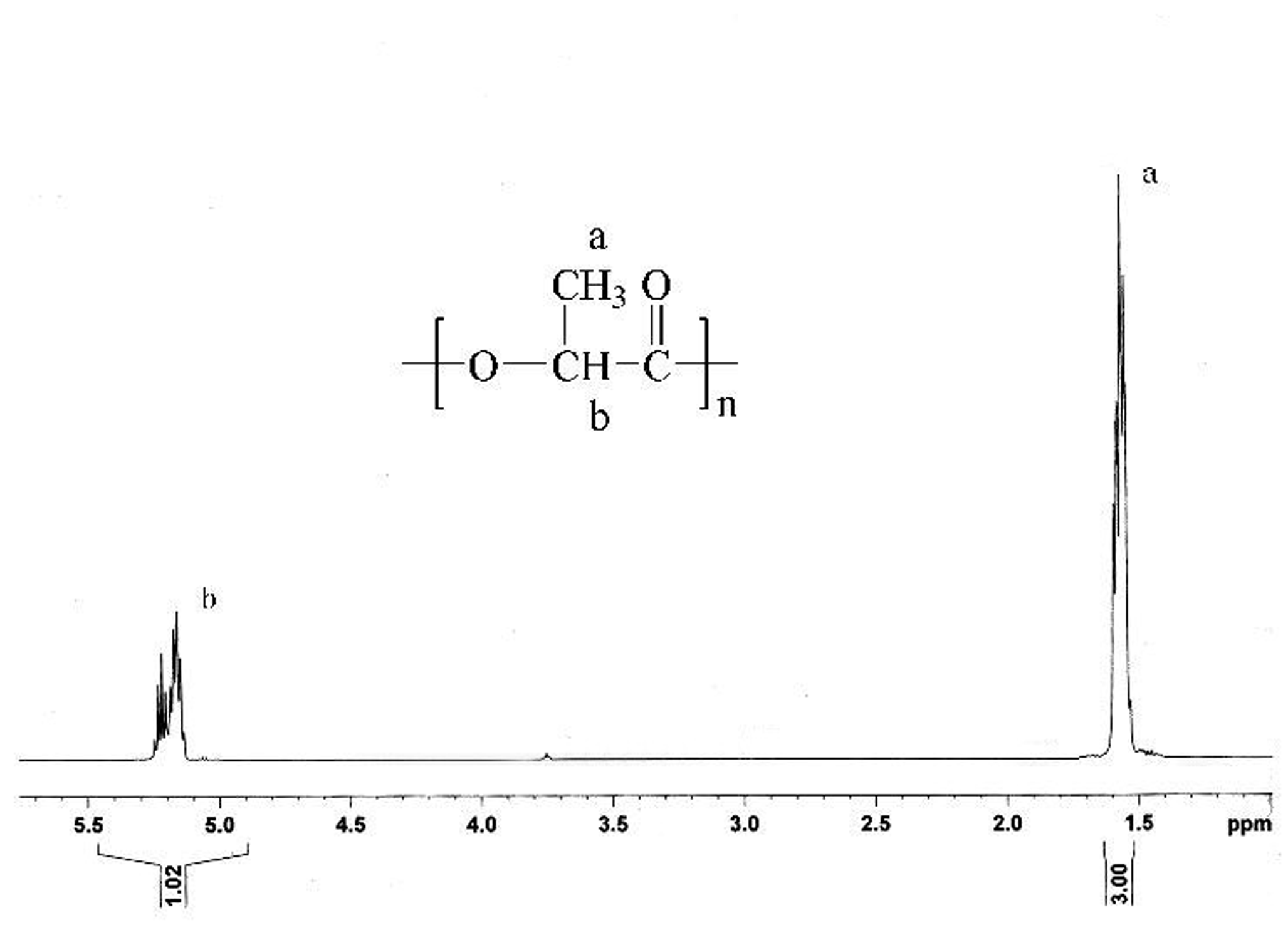

Supplement: S3 Fig — (TIF) [file pone.0201054.s004.tif]

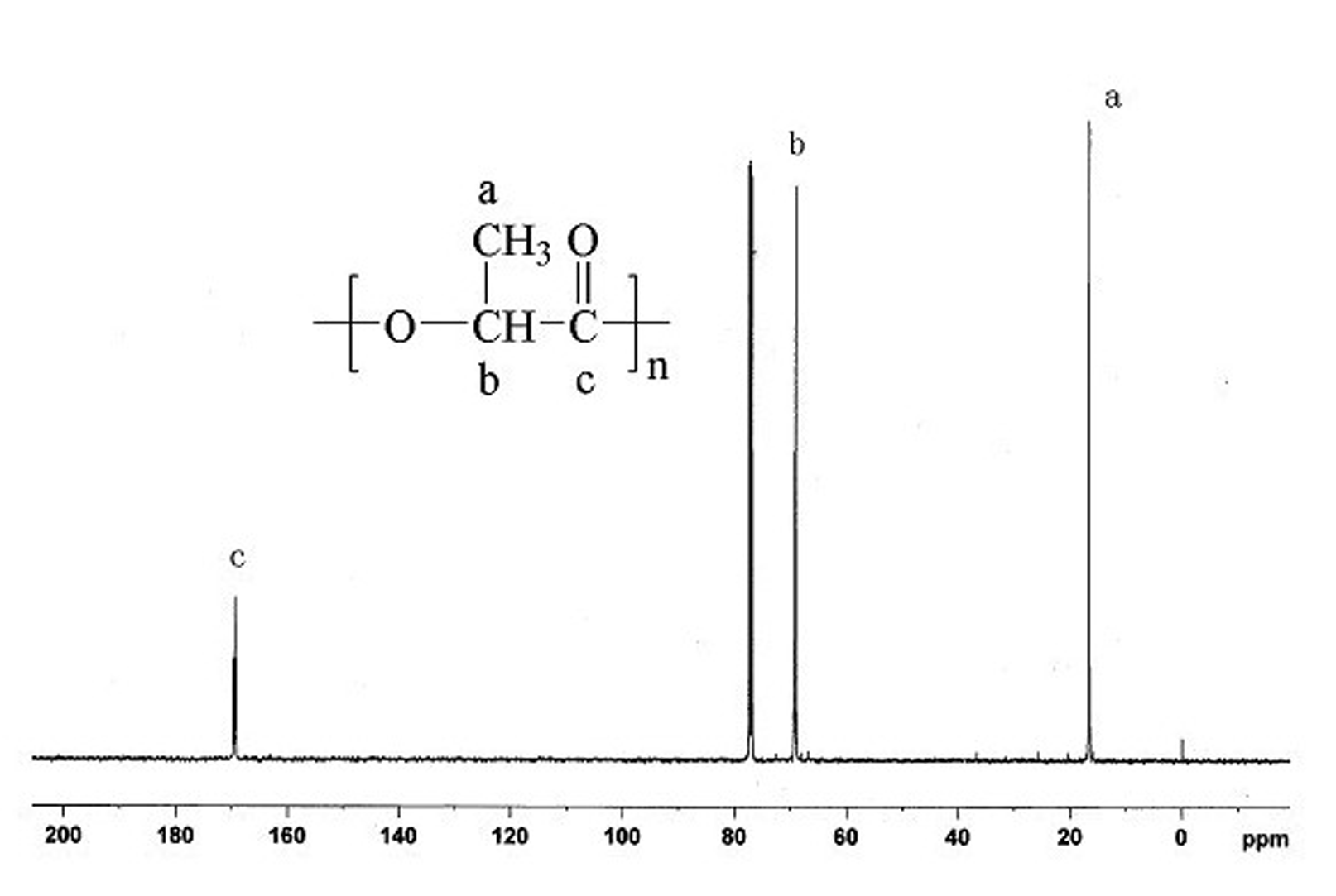

Supplement: S4 Fig — (TIF) [file pone.0201054.s005.tif]
